# Deformable discoidal polymeric nanoconstructs: Design principles, therapeutic applications, and translational perspectives

**DOI:** 10.1007/s10544-026-00854-6

**Published:** 2026-09-22

**Authors:** Raffaele Spanò, Roberto Palomba, Alessia Felici, Paolo Decuzzi, Anna Lisa Palange

**Affiliations:** 1 Laboratory of Nanotechnology for Precision Medicine, Fondazione Istituto Italiano di Tecnologia, Genoa, 16163 Italy; 2 Division of Oncology, Department of Medicine, Department of Pathology, Stanford University School of Medicine, Stanford, CA 94305 USA

**Keywords:** Deformable nanoconstructs, Drug delivery, Particle mechanics, Cancer nanomedicine, Thrombolytic therapy

## Abstract

Deformable Discoidal Polymeric Nanoconstructs (DPN) represent a biomimetic drug-delivery platform designed to overcome the limitations of conventional spherical nanoparticles. Their discoidal geometry, tunable deformability, and PLGA/PEG-based composition promote vascular margination, reduce phagocytic sequestration, prolong circulation, and enhance accumulation at pathological sites. This review highlights the design principles, fabrication strategies, therapeutic applications, and translational potential of DPN. In oncology, DPN have been engineered to improve the loading and sustained release of docetaxel through multi-passage fabrication, prodrug conjugation, and hierarchical micro-combinatorial hydrogel particles (µCGP). These discoidal particles enhanced therapeutic efficacy in preclinical models of triple-negative breast cancer, glioblastoma, and lung metastases. Beyond cancer, surface-functionalized DPN carrying tissue plasminogen activator improved clot targeting and thrombolysis while limiting neurological toxicity in ischemic stroke models. Overall, DPN integrate geometry, mechanics, and surface functionality to achieve vascular confinement, targeted delivery, and improved safety. Their modular design, biocompatible materials, and favorable preclinical performance support further development as versatile platforms for cancer therapy, metastatic disease, and thrombotic disorders.

## Deformable discoidal polymeric nanoconstructs (DPN): An introduction

Despite the remarkable progress made in nanomaterial science over the past two decades, translating nanoparticle-based drug delivery systems into effective clinical tools has proven far more challenging than initially anticipated. Conventional carriers, predominantly spherical polymeric nanoparticles and liposomes in the 100–200 nm size range, have been refined through successive generations of surface engineering, including PEG modifications, corona formation, ligand-mediated active targeting, and stimuli-responsive payload release. Nevertheless, the therapeutic performance of conventional carriers at the tumor site continues to fall short of expectations (Palange et al. 2017). A landmark meta-analysis of the field estimated that, on average, less than 1% of an administered nanoparticle dose actually reaches solid tumor tissue (Wilhelm et al. 2016), a sobering figure that has prompted serious reconsideration of the prevailing design paradigms. While PEGylation of biodegradable polymer matrices such as PLGA does confer meaningful improvements in circulatory persistence by partially shielding carriers from opsonization and mononuclear phagocyte system recognition, this strategy alone has proven insufficient to overcome the complex biological barriers existing between the injection site and the tumor microenvironment (Danhier et al. 2012). Recently, the use of machine learning models is emerging as a powerful approach to enable a more rational design of polymeric spherical nanocarriers (Basak 2026; Francesco et al. 2023).

To overcome the common limitation of the polymeric spherical nanocarriers, Prof. Paolo Decuzzi and colleagues have developed a conceptually distinct class of particles: Deformable Discoidal Polymeric Nanoconstructs (DPN), whose architecture deliberately moves away from the spherical norm and instead draws its logic from the physical and mechanical properties of circulating blood cells (Key et al. 2015; Palomba et al. 2018; Ferreira et al. 2020; Felici et al. 2022; Spanò et al. 2025). Table 1 compares the key features of DPN, including drug loading and circulation half-life with those of conventional spherical PLGA nanoparticles and liposomes.

**Table 1:** Comparison between PLGA polymeric systems and liposomes. Performance parameters are representative benchmark values

Feature	DPN	Conventional spherical PLGA nanoparticles	Liposomes
Typical structure/geometry	Discoidal, typically ~1,000 x 400-500 nm; geometry and stiffness independently tunable	Predominantly spherical; commonly ~50-300 nm	Spherical phospholipid bilayer vesicles; commonly ~50-200 nm
Drug-loading performance	Strongly payload-and method-dependent; Absorption loading produced encapsulation efficiencies up to ≈ 80% for moderately hydrophobic, low-MW compounds; Sirect loading was generally <1% in the reported DPN system	Highly formulation-dependent; Hydrophobic drugs are generally incorporated more efficiently than hydrophilic molecules; Loading controlled by polymer/drug ratio, solvent system and fabrication method	Highly formulation-dependent; Passive loading may be limited for some hydrophilic drugs, whereas active/remote loading can produce >90-95% EE for suitable ionizable drugs such as doxorubicin
Representative circulation half-life	Soft DPN: ≈ 24h in tumor-bearing mice	Unmodified PLGA NPs can clear relatively rapidly: one mouse study reported ≈ 45-51 min.; Pegylated PLGA-based systems can reach several hours depending on PEG density and particle size	PEGylated liposomal formulations are long-circulating. Liposomal doxorubicin shows approximately 20-30h terminal half-life in preclinical studies, with susbstantially longer terminal phases also reported clinically
Manufacturing method	Top-down, template-based fabrication: lithographically defined master/template, with a sacrificial PVA mold. PLGA/PEG-DA polymer paste filling, with PEG-DA cross-linking . Final step with template dissolution and particle recovery	Usually bottom-up methods such nanoprecipitation, single/double emulsion-solvent evaporation or solvent diffusion	Lipid self-assembly, commonly thin-film hydration, ethanol-injection methods or microfluidic mixing, often followed by extrusion; Active drug loading can be performed after vescicle formation
Distinctive advantage	Precise independnt control of shape, size and mechanical stiffness, with geometry designed to influence margination, phagocytic uptake and vascular interactions	Established biodegradable polymer platform with relatively simple formulation and broad payload compatibility	Clinically mature platform; Excellent biocompatibility; Ability to accommodate both hydrophilic and lipophilic drugs
Major limitation	More complex template-based fabrication and scale-up than conventional self-assembled nanoparticles	Limited independent control of particle geometry; Rapid MPS clearance without appropriate surface modification	Stability; Leakage; Lipid oxidation/hydrolysis; Formulation-dependent loading; Manufacturing requires tight control of lipid composition and size

### Design rationale and biological inspiration

The design philosophy underpinning DPN is rooted in a careful study of erythrocyte behavior in the vascular compartment. Native red blood cells evade immune clearance through a combination of two main characteristics: (i) surface protein expression and (ii) geometric deformability. The first has inspired multiple research groups to physically cloak conventional PLGA cores with membrane material harvested directly from red blood cells (Hu et al. 2011; Ben-Akiva et al. 2020). The second, purely focusing on geometric deformability inspired the conceptualization of DPN and other non-spherical particles. For instance, the Particle Replication in Non-wetting Templates (PRINT) technology developed by DeSimone and colleagues enables the fabrication of monodisperse polymeric particles with precisely controlled size, shape, composition, and deformability, including cylindrical, filamentous, and discoidal geometries (Rolland et al. 2005; Kersey et al. 2012; Perry et al. 2012). On the other end, Discher and colleagues developed flexible filamentous micelles, or filomicelles, to mimic the elongated morphology and flow behavior of filamentous biological structures, demonstrating prolonged circulation and reduced cellular uptake compared with spherical counterparts (Geng et al. 2007).

All these complementary approaches collectively demonstrate that geometry, deformability, and surface biomimicry represent distinct but potentially synergistic design strategies for controlling the biological fate of nanocarriers.

The hemodynamic rationale for the discoidal shape is well established in the literature: non-spherical particles, and flat disc-shaped ones in particular, interact more favorably with the RBC-depleted plasma layer adjacent to the vessel endothelium, a phenomenon known as margination (Alexis et al. 2008). Unlike spheres, which tend to remain centrally suspended in flowing blood, particles with a high aspect ratio and flat profile are subject to asymmetric hydrodynamic forces that push them laterally toward the vessel wall, a prerequisite for any subsequent adhesion or drug release at the endothelial surface. Computational fluid dynamics studies have confirmed that margination efficiency improves with increasing particle size and with departure from spherical symmetry, as the reduced rotational freedom of flat particles near a surface prolongs their wall-proximal residence time (Müller et al. 2014). These physical principles directly informed the choice of a disc-shaped architecture as the structural basis for DPN, an approach that had already been validated in early tumor imaging studies from the same group (Key et al. 2013).

### Fabrication and material composition

**Fig. 1:**
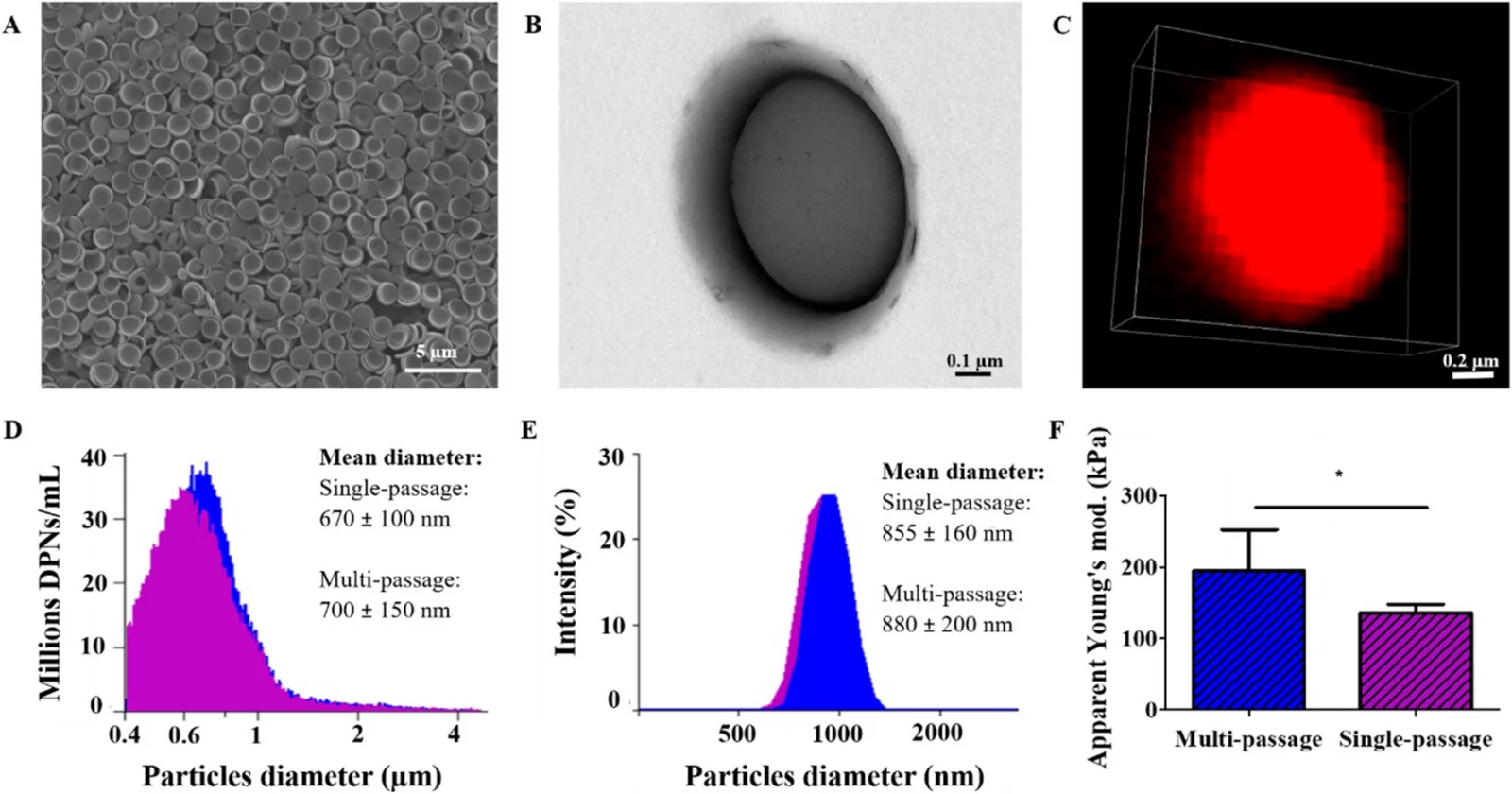
DPN morphological and mechanical characterizations.(**A**) Scanning electron microscopy images of DPN (**B**) Electron microscopy of an individual, tilted DPN; (**C**) Fluorescent microscopy of a DPN carrying Rhodamine B molecules; (**D**) Multisizer Coulter Counter profile of single- and multi-passage DPN; (**E**) Dynamic light scattering size distribution for single- and multi-passage DPN; (**F**) Young’s modulus under compression for single- and multi-passage DPN. *From: Felici A. et al.*,* Vascular-confined multi-passage discoidal nanoconstructs for the low-dose docetaxel inhibition of triple-negative breast cancer growth*, https://doi.org/10.1007/s12274-021-3507-8, *licensed under CC BY 4.0*

A fundamental advantage of DPN over most competing nanomedicine platforms lies in the precision of their fabrication process. Rather than relying on thermodynamic self-assembly (the basis of emulsification, nanoprecipitation, and solvent displacement methods, all of which produce predominantly spherical particles with limited independent control over geometry) DPN are produced through a top-down, template-assisted manufacturing strategy (Key et al. 2013). The critical consequence of this workflow is that particle diameter, height, aspect ratio, and mechanical stiffness can each be tuned by modifying the template geometry or the paste composition, without altering any other particle property (Key et al. 2015). In terms of chemistry, the structural matrix of DPN is formed by the physical entanglement and crosslinking of PLGA and PEG chains, yielding a hydrogel-like composite whose degradation proceeds through the well-characterized hydrolytic breakdown of the ester bonds in PLGA into lactic and glycolic acid monomers, metabolites that are cleared through normal physiological pathways and that have an extensively documented safety profile in humans (Kapoor et al. 2015). The selection of PLGA as the principal polymer component places DPN within a well-established regulatory framework, given its use in several FDA-approved drug delivery products and medical devices. Importantly, the modular nature of the fabrication platform is not chemically restrictive: the polymer paste can in principle incorporate several components, enabling straightforward adaptation of the platform to different needs (Ferreira et al. 2020). What genuinely distinguishes DPN from the large body of PLGA-based spherical nanoparticle research (Danhier et al. 2012; Kapoor et al. 2015) is therefore not the choice of materials per se, but the geometric and mechanical precision that the template-based approach uniquely affords a dimension of design that conventional bottom-up formulation methods cannot access (Fig. 1).

### Mechanical stiffness as a design parameter

Perhaps the most conceptually novel aspect of DPN engineering is the treatment of mechanical stiffness as an independently tunable variable, rather than an incidental consequence of material selection. By systematically varying the ratio of PLGA and PEG in the polymer paste while holding all other fabrication parameters constant, the Decuzzi group produced DPN variants spanning from soft constructs with a Young’s modulus of approximately 1.3 kPa, to rigid ones at around 15 kPa, all sharing the same 1,000 × 400 nm disc geometry and a surface zeta potential of approximately − 14 mV^7^. This approach transforms stiffness into a design handle with direct consequences for immune evasion and circulatory behavior. The broader nanomedicine literature has increasingly converged on the view that particle deformability profoundly influences interactions with phagocytic cells. Mechanistic studies using liquid-filled silica nanocapsules of calibrated stiffness have shown that softer particles resist macrophage internalization significantly more effectively than their rigid counterparts, a difference attributed to the energetic cost of membrane wrapping around a deformable object, which requires more work than engulfing a geometrically stable, hard particle (Hui et al. 2019). Quantitatively, macrophage uptake of soft nanocapsules has been found to be reduced by approximately threefold relative to stiff equivalents of comparable size and surface chemistry (Anselmo and Mitragotri 2017). These findings align with thermodynamic modeling, which predicts that the energy barrier to complete phagocytic engulfment rises as particle compliance increases, since a deformable particle redistributes membrane tension during wrapping in a way that penalizes complete internalization. Within the DPN framework, Palomba, et al., translated these principles into a systematic experimental study spanning multiple particle geometries: circular, quadrangular, and elliptical; across a stiffness range from approximately 100 kPa to 10 MPa, confirming that reduced Young’s modulus consistently and substantially lowered phagocytic uptake regardless of particle shape (Palomba et al. 2018). Crucially, this immune evasion is achieved through purely synthetic means by tuning crosslink density in the polymer network, conferring DPN with a reproducibility and scalability advantage that becomes increasingly important as these constructs move toward potential clinical development.

### In vivo behavior: Circulation, tumor targeting, and imaging

The synergistic combination of disc-shaped geometry and mechanical softness is expected to improve the in vivo pharmacokinetic behavior of DPN. In preclinical studies using tumor-bearing mouse models, including brain and skin cancer xenografts, intravenously administered soft DPN maintained detectable blood concentrations for approximately 24 h post-injection, and achieved tumor tissue accumulation of up to 20% of the injected dose per gram of tumor (Key et al. 2015). This figure is striking in the context of the field-wide average of below 1% ^2^, and is all the more noteworthy because it is accomplished passively, without active targeting ligands, relying entirely on the physical properties of the construct to navigate away from the mononuclear phagocyte system and toward the tumor vasculature. The mechanistic basis for this preferential accumulation lies in the structural characteristics of tumor microvessels: their irregular caliber, high tortuosity, and sluggish perfusion collectively create flow conditions under which flat, deformable particles are more readily arrested and retained at the vascular wall than spherical ones (Ferreira et al. 2020). Taken as a whole, the physical, chemical, and biological properties reviewed in this section converge to define a nanocarrier whose performance advantages over conventional spherical platforms are not incidental but structurally built in. The sections that follow examine how these advantages have been translated into concrete preclinical outcomes in oncology and cerebrovascular disease.

## Deformable discoidal polymeric nanoconstructs for cancer therapy: Exploring different strategies to boost therapeutic effectiveness

Non-spherical nanoparticles have enabled new strategies to overcome biological barriers that limit nanoparticles’ accumulation in solid tumors that are difficult to treat with conventional therapies, including breast, lung, and brain cancers (Decuzzi et al. 2010).

By leveraging their unique morphological properties, DPN are optimized to ensure a slow and sustained release of chemotherapies within the blood circulation, facilitating the suppression of circulating tumor cells responsible for metastasis and regression of tumor mass.

These optimizations are driven by previous studies that established the enhanced efficacy of DPN relative to conventional nanoparticles for the co-delivery of multimodal diagnostic agents including DSPE-DOTA, DSPE-Rhodamine, and iron oxide nano cubes (Palange et al. 2017, Key et al. 2015). The remarkable results were mainly attributed to the discoidal shape and the spongy structure of the PLGA- PEG-diacrylate matrix that drastically reduced recognition by the mononuclear phagocyte system (MPS) (Palomba et al. 2018).

Although DPN offer favorable vascular transport and mechanical properties, initial formulations exhibited limited retention of small chemotherapeutic molecules. This limitation is primarily attributed to weak chemical drug-polymer interactions and the purification steps of the top-down fabrication process.

It is important to emphasize that the deposition of PLGA chains differs substantially from that observed in nanoprecipitation, where strong hydrophobic interactions drive the system toward more compact architecture. While this top-down approach enables high versatility in the mechanical control of nanofabrication, it inherently demands drug loading optimization.

The mechanical softness required to minimize macrophage uptake and prolong blood circulation comes at the cost of poor retention for small molecules (500-1’000 Da) within the PEG-cross-linked polymer chains. This issue was bypassed by the contrast agents utilized in previously cited biodistribution studies, which were either lipid-conjugated or comprised of nanoscale iron oxide nanocubes (5–20 nm).

We addressed this limitation using three different approaches: first, by increasing the number of deposited polymer layers, a strategy defined “multi-passage” approach (Felici et al. 2022); second, by modulating the interactions between PLGA and chemotherapeutics through the synthesis and testing of a series of docetaxel prodrugs (Felici et al. 2022); and third, by engineering a multi-compartment technology (Palange et al. 2023). The first strategy demonstrated a two-fold improvement in docetaxel loading efficiency within DPN using a ‘multi-passage’ approach, wherein multiple polymer deposition layers were applied without altering the PLGA-to-PEG ratio.

This method enabled greater drug entrapment and retention in the nanostructure, producing more uniform and well-defined particles. Consequently, the therapeutic performance of docetaxel-DPN (DTXL-DPN) in an orthotopic murine model of triple-negative breast cancer (TNBC) showed significantly higher efficacy in vivo delivering more chemotherapy agents compared with the free docetaxel administered intravenously. The results were encouraging, demonstrating therapeutic efficacy at a low dose of 3 mg/kg in an aggressive model of TNBC; however, it is important to note that the multi-passage approach inevitably leads to well defined structures with increased rigidity (Fig. 1). Therefore, other approaches were explored to balance mechanical properties and drug retention.

In contrast, the second approach was designed not only to enhance the loading of small molecules but, primarily, to modulate the release rate of docetaxel. Leveraging insights from the prior research utilizing lipid-conjugates for contrast agents’ integration, a series of four docetaxel prodrugs were synthesized. The release kinetics of molecules loaded within the PLGA matrices are governed not only by polymer backbone hydrolysis but most importantly by drug-related parameters such as solubility, size and chemical interactions with the matrix, as supported by coarse-grained molecular dynamic simulations (Pannuzzo et al. 2022).

Although the oleic-docetaxel conjugate (OA-DTXL) exhibited a promising encapsulation efficiency of 56% while forming compact nano-sphere on the surface of DPN, its release kinetics was excessively slow, with only 10% of docetaxel released after 72 h. A similar trend was observed for the prodrugs bearing the longest PEG chain (PEG_1000_-DTXL), which formed nanosphere within the matrix and showed about 50% release at 72 h, yet with encapsulation efficiency lower than that of free DTXL.

**Fig. 2:**
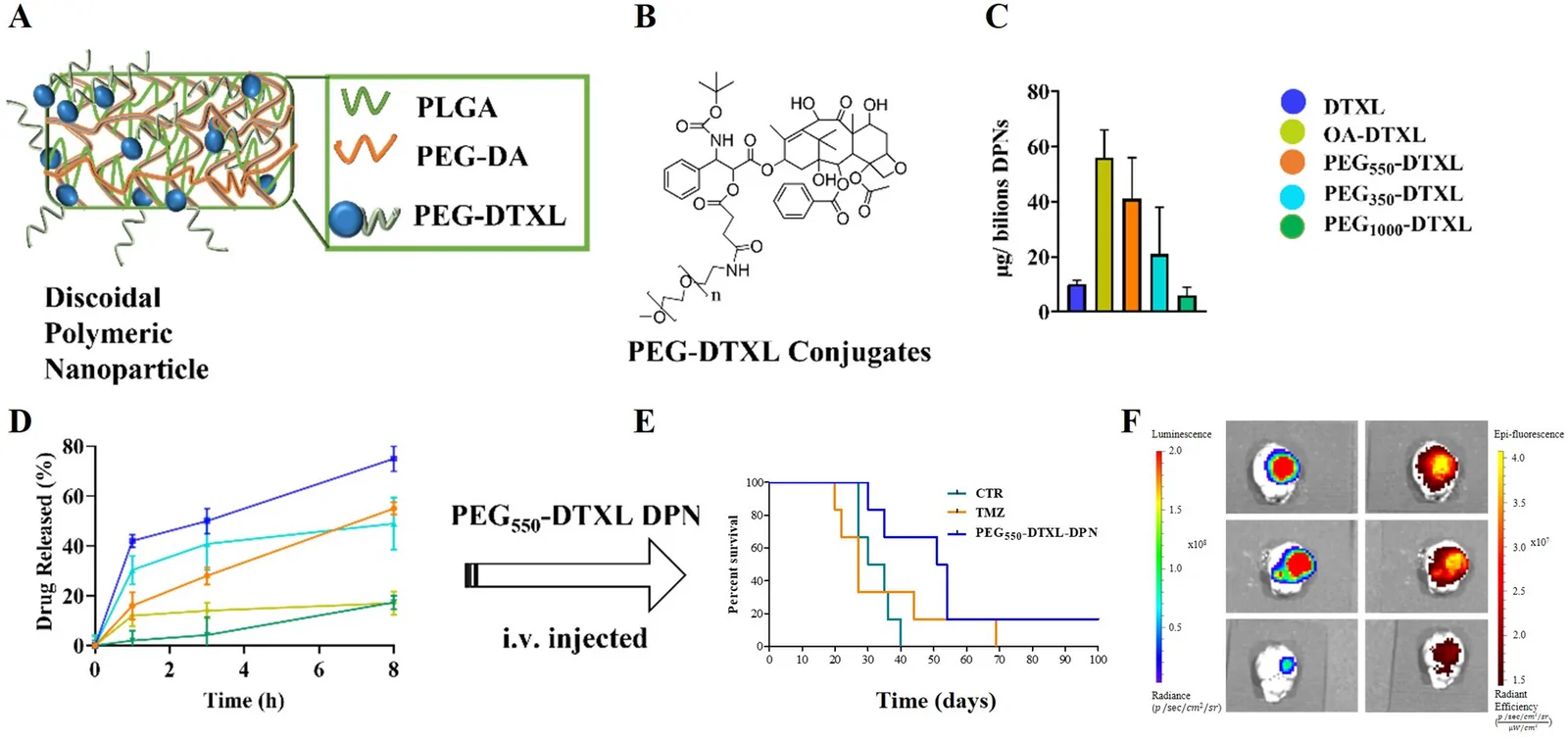
Pharmaceutical and therapeutic performance of DTXL-DPN.(**A**) Schematic representation of DPN loaded with a DTXL prodrug. (**B**) PEG-docetaxel prodrug chemical structures. (**C**) Encapsulation efficiency of DTXL, OA-DTXL and PEG-DTXL complexes in DPN expressed as mass of DTXL per billion DPN, with 10 mg/ml drug inputs (*n* = 3). (**D**) Release profile of DTXL and PEG-DTXL complexes from DPN under infinite sink conditions (pH = 7.4 and 37° C) (*n* = 3). (**E**) Kaplan–Meier curves for overall survival for the three experimental groups (Green – saline, CTR; Orange: free TMZ; Blue: PEG_550_-DTXL-DPN). (**F**) Ex-vivo imaging (BLI, left) and fluorescence (FLUO, right) analyses of the brain tumors harvested at 24 h post Cy5-DPN injection. The BLI signal is associated with the malignant mass, whereas the FLUO signal is related to the Cy5-DPN accumulation. *Adapted from: Felici A. et al.*,* Boosting the therapeutic efficacy of discoidal nanoconstructs against glioblastoma with rationally designed PEG-docetaxel conjugates*,* doi*: https://doi.org/10.1016/j.ejpb.2022.03.011, *licensed under CC*

In contrast, intermediate hydrophilic PEG derivatives (PEG_350_/PEG_550_) showed promising therapeutic performance in vitro and the lead candidate, PEG_550_-DTXL, has been further investigated in a model of glioblastoma (Fig. 2). Notably, short PEG chains, below 1 kDa, allowed the authors to chemically modify docetaxel without affecting its cytotoxic efficacy at longer time points, 72 h and beyond. Indeed, while the hydrophobic DTXL can rapidly enter the cell and exert its killing activity, the heavier and less hydrophobic PEG-DTXL conjugate exhibits a delayed entry. Ultimately, after a sufficiently long incubation period, the conjugate yields IC_50_ values comparable to the parent drug.

In vivo, in a preclinical orthotopic murine model of Glioblastoma (GBM), the treatment with PEG_550_-DTXL-DPN slowed down significantly the progression of the disease as compared to Telozolomide (TMZ) and untreated mice (saline, CTR). No untreated mice survived beyond 40 days. Similarly, 80% of TMZ-treated mice had to be sacrificed before day 40, whilst only 40% of PEG_550_-DTXL-DPN treated mice succumbed before 40 days. This is more clearly presented via the Kaplan–Meier curves confirming that all the CTR were sacrificed within 40 days of post study initiation (Fig. 2E). In conclusion, these works demonstrate that DPN can be engineered to deliver hydrophobic compounds, such as free DTXL, as well as prodrugs like PEG-conjugates of DTXL with sustained release profile and enhanced therapeutic efficacy when compared with the parent drug.

## Micro-combinatorial hydrogel particles for lung metastasis treatment

Inspired by the works published in 2015 on ACS Nano (Key et al. 2015), 2022 on Nano Research (Felici et al. 2022) and 2023 on European Journal of Pharmaceutics and Biopharmaceutics (Felici et al. 2022) a new class of non-spherical nanoconstructs was designed. Previous research has extensively demonstrated DPN high tumor accumulation and long circulation half-life (Key et al. 2015) while also highlighting some limitations, such as the low loading capacity and poor retention of small molecules. Both issues, associated with the spongy nature of the polymeric matrix have only been partially addressed with the two strategies described above focusing on the development of multi-passage DPN and prodrugs loaded DPN (Felici et al. 2022). A third restriction linked with DPN geometry regards the modest tissue permeation.

**Fig. 3:**
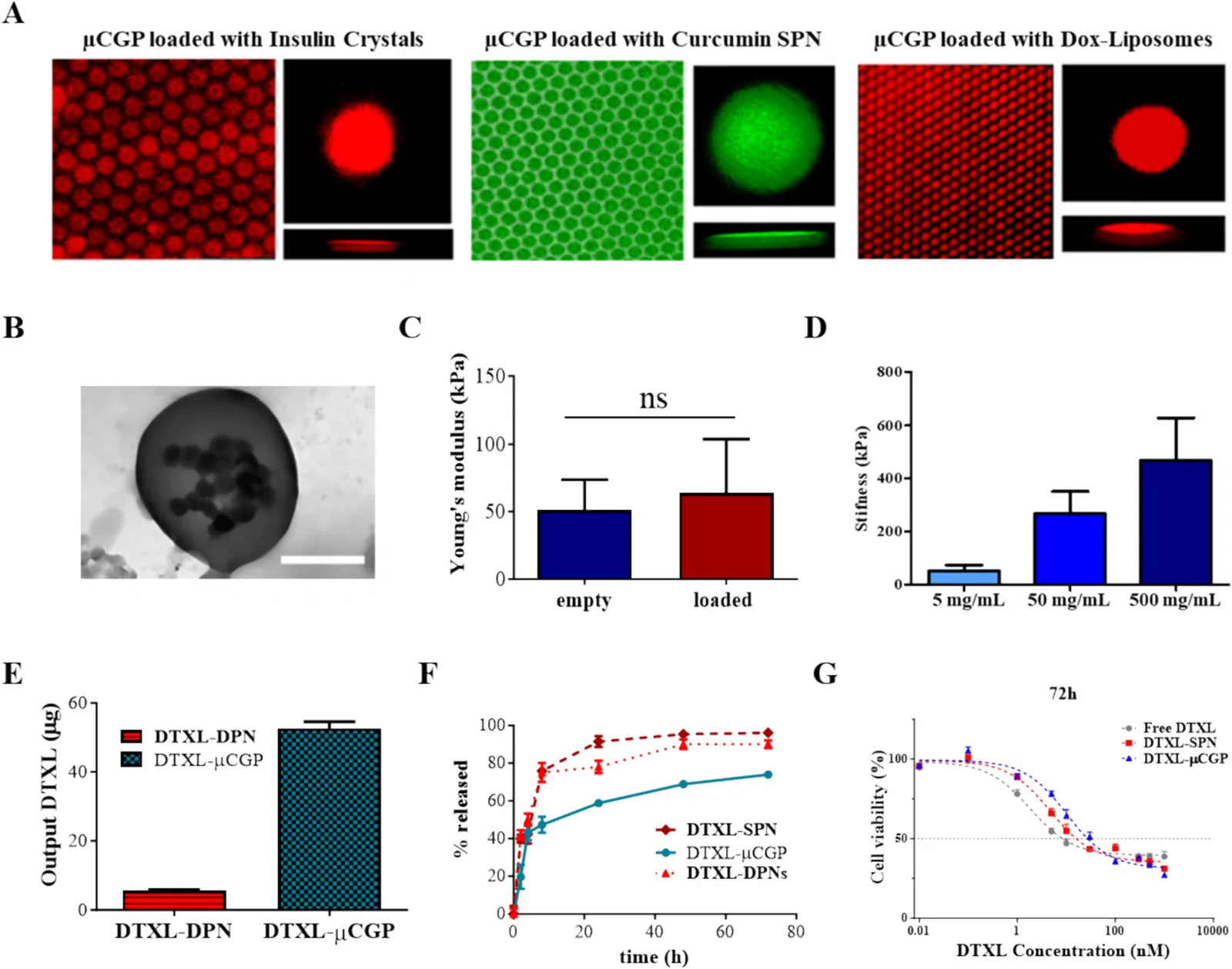
Micro-Combinatorial Hydrogel Particles(**A**) Confocal images showing µCGP incorporating insulin crystals, curcumin-loaded SPN and doxorubicin (DOX)-loaded liposomes; (**B**) TEM image showing uniform distribution of SPN within the µCGP matrix; (**C**) Apparent Young’s modulus for the empty and loaded of µCGP, as derived from the AFM indentation curves; (**D**) Apparent Young’s modulus for µCGP synthetized with increasing amounts of PEGDA; (**E**) Drug loading into µCGP and DPN; (**F**) Drug release from DTXL-SPN, DPN and DTXL-µCGP up to 72 h (PBS, 37 °C, 4 L); (**G**) Cell viability studies for MDA-MB-231 cells treated with free DTXL, DTXL-SPN, DTXL-µCGP for 72 h. *Adapted from: Palange A. L. et al.*,* Boosting the Potential of Chemotherapy in Advanced Breast Cancer Lung Metastasis via Micro-Combinatorial Hydrogel Particles*,* doi*: https://doi.org/10.1002/advs.202205223, *licensed under CC BY 4.0. Modifications include rearrangement*,* cropping*,* and combination of original plots.*

To overcome these obstacles, micro Combinatorial Hydrogel Particles (µCGP), have been developed (Palange et al. 2023). µCGP is composed of 2 μm discoidal hydrolytically labile PEG network encapsulating small nanoparticles (~ 200 nm) and a variety of smaller agents, including molecules, exosomes, and antibodies, each providing a specific imaging or therapeutic function. In this system, the discoidal PEG hydrogel behaves similarly to DPN, accumulating in the perivascular region. Given the hydrolytically labile nature of the PEG network, the smaller payloads are gradually released and can penetrate deep into the tumor tissue. Notably, µCGP combine the advantages of DPN and highly permeable small nanoparticles. A key aspect of this development was optimizing the top-down fabrication method by eliminating the use of organic solvents. This modification was essential, as the presence of solvents would have prevented efficient loading of nanoparticles within the hydrogel mesh. Consequently, one of the main advantages of this platform is its reliance on a solvent-free fabrication approach. A second notable feature of this platform is its capacity to enable combinatorial therapeutic strategies, as the discoidal backbone can simultaneously accommodate nanoparticles and/or small molecules targeting distinct biological pathways (Fig. 3A). In support of this capability, Fig. 3A illustrates the successful incorporation of diverse nanoparticle systems within µCGP, including insulin crystals for diabetes management, curcumin-loaded SPN for anti-inflammatory applications, and doxorubicin (DOX)-loaded liposomes for cancer therapy.

A critical aspect in the design of µCGP was the evaluation of the mechanical stiffness. Being a hydrogel, µCGP exhibit a lower stiffness compared to DPN. Importantly, the incorporation of small nanoparticles does not significantly alter the mechanical properties of the system (Fig. 3B-C). Conversely, the stiffness of µCGP can be finely tuned by modulating the polymer concentration, which in turn enables control over the release kinetics of the encapsulated cargo (Fig. 3D). From a pharmacological perspective, µCGP demonstrate significantly improved loading capacity and release profiles compared to DPN, thereby addressing one of the main limitations of the latter while preserving the network deformability, a key feature for systemic applications (Fig. 3E-F). The first application for µCGP concerned the treatment of lung metastasis originating from triple-negative breast cancer (TNBC). To this end, µCGP have been loaded with spherical polymeric nanoparticles carrying docetaxel (DTXL). This study published in Advanced Science (Palange et al. 2023) confirms the dual behavior of µCGP, with the PEG backbone predominantly localizing at the cell surface and the small NP progressively being released and penetrating tumor tissue. The in vitro therapeutic efficacy was evaluated in MDA-MB231 triple negative breast cancer cells. As expected, a slight delay in the therapeutic response was observed as compared to the treatment with the free chemotherapeutic and the drug loaded within the nanoparticles. The delay is attributable to the time required for cargo release. Nevertheless, IC_50_ and LD_50_ values remain in the nanomolar range, comparable to those of free DTXL and DTXL-loaded SPN (Fig. 3G).

**Fig. 4:**
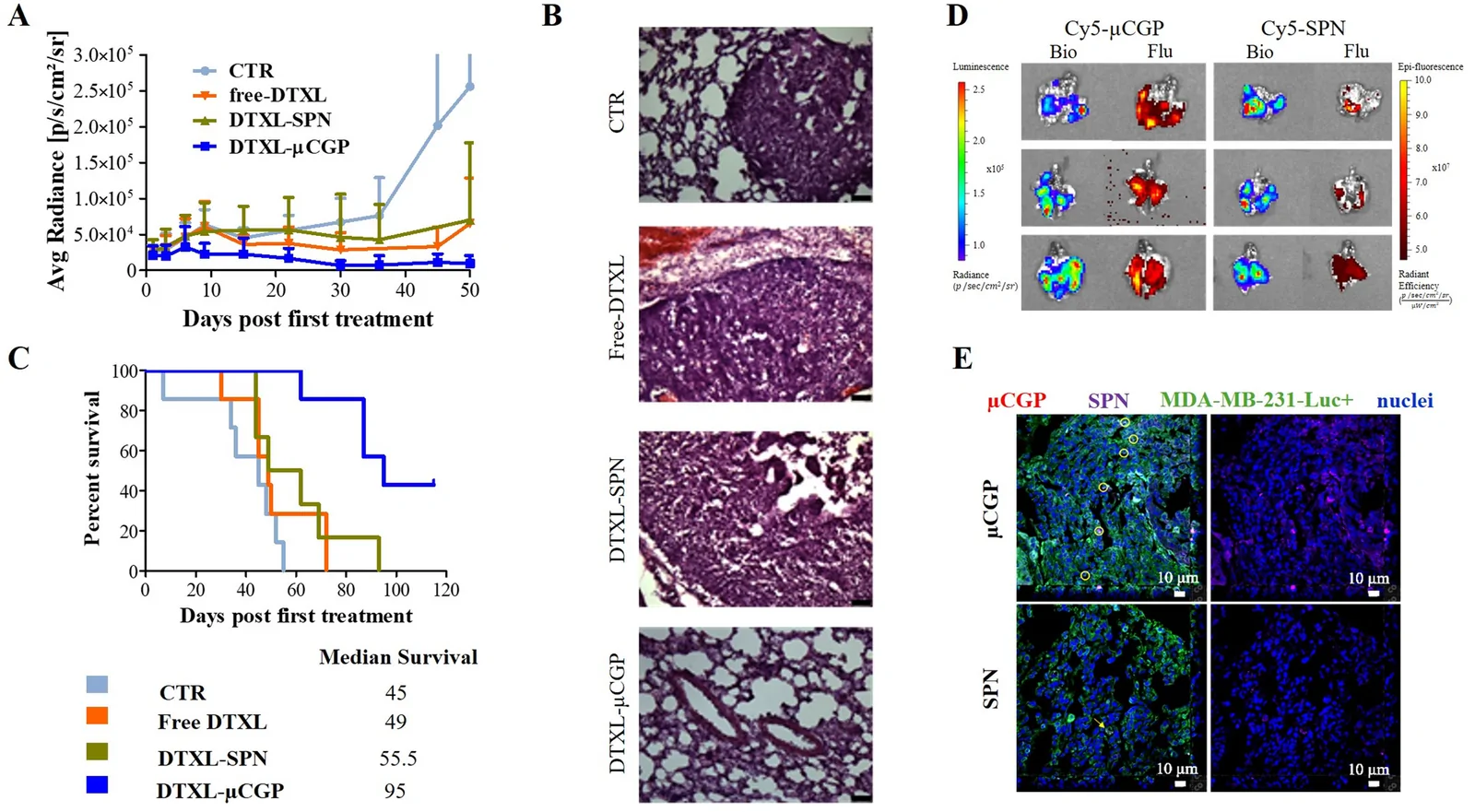
In vivo performance of Micro-Combinatorial Hydrogel Particles.(**A**) Variation of the BLI signal over time for all the mice and experimental groups; (**B**) Histological analysis by H&E staining of lungs for all the experimental groups. (Scale bar = 50 μm); (**C**) Kaplan–Meier survival curves and list of median survivals; (**D**) ex vivo bioluminescence (left) and fluorescence (right) images of the main organ (left) and lung nodules (right) at 24 h post injection of µCGP loaded with Cy5-SPN. (**E**) Confocal fluorescent images of lung nodules at 24 h post injection of either Cy5-µCGP loaded with RhB-SPN (µCGP) or freely administered RhB-SPN (SPN). *Adapted from: Palange A. L. et al.*,* Boosting the Potential of Chemotherapy in Advanced Breast Cancer Lung Metastasis via Micro-Combinatorial Hydrogel Particles*,* doi*: https://doi.org/10.1002/advs.202205223, *licensed under CC BY 4.0. Modifications include rearrangement*,* cropping*,* and combination of original plots.*

For in vivo evaluation, µCGP loaded with DTXL-SPN were tested in a late-stage lung metastasis model, in which treatment was initiated one month after tumor cell inoculation. The discoidal geometry of µCGP confers a preferential accumulation within the complex lung capillary network. Therapeutic efficacy was significantly enhanced compared to both free drug and standalone nanoparticles, as evidenced by radiance measurements, and histological analyses (Fig. 4A-B). Notably, lung tissue from µCGP -treated animals exhibited preservation of the alveolar architecture, whereas control groups showed substantial disruption due to metastatic nodule expansion. Survival analysis further highlights the therapeutic benefit, with approximately 50% of animals treated with DTXL-µCGP surviving up to four months post-treatment initiation (five months post-inoculation), whereas all control groups succumbed within three months (Fig. 4C). Imaging studies supported the in vivo therapeutic outcome. IVIS imaging data indicate substantial lung accumulation and colocalization of µCGP within lung tissue, while minimal accumulation is observed for SPN alone (Fig. 4D). Finally, confocal imaging of lung sections provides additional evidence of the advantages of this hierarchical delivery system. A markedly enhanced diffusion of SPN (indicated by the purple signal) is observed in µCGP -treated animals, whereas minimal SPN accumulation is detected in metastatic nodules when administered alone (Fig. 4E). Overall, these results support the notion that the enhanced permeability and retention (EPR) effect is not a dominant mechanism in metastatic disease and that given their flexible design and modular architecture, µCGP could become promising delivery platforms for treating metastatic disease. Despite this encouraging outlook, the regulatory development of µCGP is expected to be particularly challenging. The clinical translation of complex, multi-compartment platforms may extend across conventional regulatory categories, especially when drug, device, and biological components are integrated within a single product. Under both FDA and EMA frameworks, such systems may be classified as combination products, with the regulatory pathway generally determined by their primary mode of action. However, the increasing number and complexity of constituent components can complicate product classification, manufacturing controls, preclinical assessment, and the demonstration of safety and efficacy. Additional challenges include ensuring manufacturing reproducibility, product stability during storage, and a thorough understanding of the interactions between carrier materials and therapeutic payloads, as these may affect the overall safety and performance of the final product. Consequently, highly integrated delivery platforms such as µCGP will likely require tailored, product-specific regulatory strategies and early engagement with regulatory authorities to facilitate their clinical translation (Reis et al. 2022, Kim et al. 2024).

## Deformable discoidal polymeric nanoconstructs against stroke and thrombotic events

DPN technology represents a promising platform for systemic intravascular drug delivery due to its preferential localization within the vascular compartment. In addition to encapsulating therapeutic agents within their polymeric matrix, DPN can also transport biomolecules conjugated onto their surface.

A representative example of this application was proposed and investigated by Colasuonno et al. in 2018 for the targeted treatment of thrombosis (Colasuonno et al. 2018). Thrombotic events can occur in different regions of the body and may arise from multiple pathological triggers (Yu et al. 2025). Under these conditions, systemic or local intravascular administration of thrombolytic agents should be performed as rapidly as possible to restore blood flow and prevent complications, which in some cases may become severe or even fatal. The duration of vascular occlusion, together with the location of the obstruction within the vascular network (e.g., major arteries versus microcirculation), is directly associated with the severity of tissue damage.

To counteract physiological clot formation associated with inflammatory or thrombotic events and maintain vascular patency, endothelial cells synthesize and release tissue-type plasminogen activator (tPA), a highly efficient serine protease that catalyzes the conversion of plasminogen into plasmin, thereby promoting fibrin clot degradation (Levin and Zoppo 1994). Its recombinant form, Alteplase^®^, is currently the treatment of choice for acute ischemic stroke, acute myocardial infarction, and pulmonary embolism, either alone or in combination with mechanical thrombectomy procedures (Reed et al. 2023).

In 2018, Colasuonno et al. developed “armed” DPN functionalized with Alteplase^®^. In this strategy, the drug was directly conjugated onto the particle surface following particle synthesis, purification, and EDC/NHS bioconjugation reaction. The EDC/NHS activation of terminal carboxyl groups on PLGA enabled covalent coupling with accessible primary amines on alteplase. Stability studies showed minimal drug detachment over time (< 10%), indicating robust surface immobilization of tPA. At the same time, the biological activity of tPA is preserved, as demonstrated by both in vitro and in vivo experiments.

In vitro, tPA-DPN showed thrombolytic efficacy comparable to free tPA under static conditions, while under dynamic flow they achieved a 50% clot reduction within 60 min, compared with 90 min for free tPA. In vivo, in a mesenteric thrombosis mouse model, tPA-DPN recanalized 90% of occluded vessels and reduced clot size by 50% within 35 min, whereas free tPA recanalized only 40% of vessels with a 20% clot reduction.

More recently, this strategy was investigated in acute ischemic stroke (Spanò et al. 2025). Besides vascular occlusion, stroke induces secondary pathological processes, including inflammation, blood–brain barrier (BBB) disruption, edema, oxidative stress, and neuronal damage, all of which limit therapeutic efficacy. BBB impairment is particularly critical because circulating molecules, including systemically administered tPA, may penetrate the brain parenchyma and exacerbate tissue injury through neurotoxic and hemorrhagic effects (Powers 2020, Hacke et al. 2008).

In this context, conjugation of tPA onto DPN offers a significant advantage by confining the drug within the vascular compartment, thereby reducing off-target effects and improving treatment safety.

**Fig. 5:**
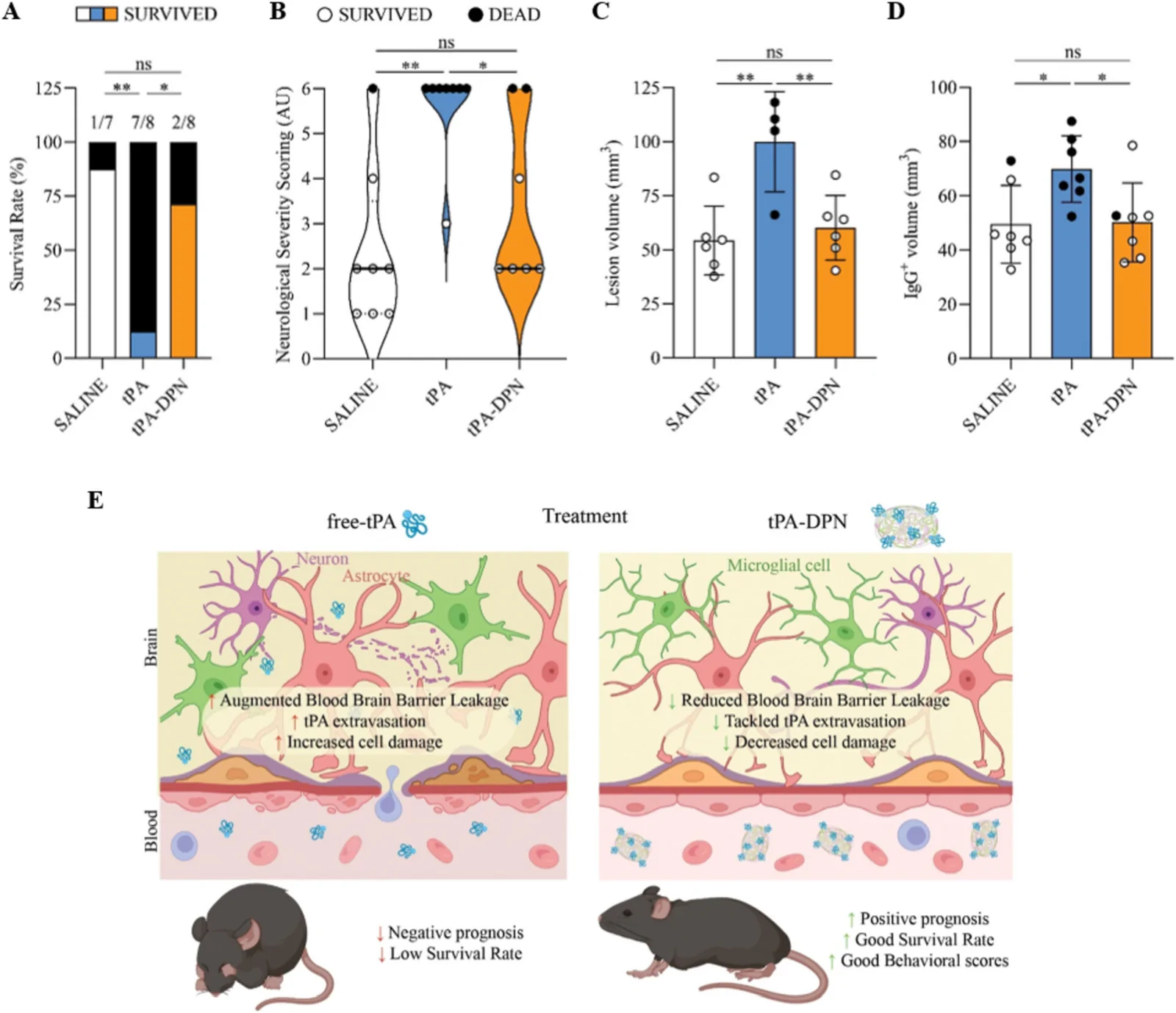
tPA-DPN enhanced safety profile of the Alteplase thrombolytic in stroke.(**A**) Survival rate 24 h post-occlusion (PS), after treatment with just vehicle (saline), free-tPA 10 mg kg^–1^, and tPA-DPN 10 mg kg^–1^. (**B**) Behavioral assessment by neurological severity scoring (NSS). (**C**) Lesion volume, expressed in mm^3^, was obtained measuring the unstained Cresyl Violet negative brain area over the sections. (**D**) IgG^+^ volume, expressed in mm^3^, was obtained measuring the brain area over the sections positive to the fluorescent IgG signal. Black symbols refer to animals found dead after 24 h. Results are expressed as mean ± SD (*n* > 4; *p < 0.05, **p < 0.01, respectively; one-way ANOVA, with Tukey correction). (**E**) Advantages of using micrometric-particles as thrombolytic agents. The intravascular confinement of tPA using tPA-microparticles, like the tPA-DPN, reduces cerebral side effects and improves survival and behavioral outcomes. *Adapted from: Spanò R. et al.*,* Enhancing Thrombolysis Safety in Post-Acute Ischemic Stroke with Tissue Plasminogen Activator-Associated Microparticles*,* doi*: https://doi.org/10.1021/acsnano.5c01499, *licensed under CC BY 4.0. Modifications include rearrangement*,* cropping*,* and combination of original plots.*

Based on these considerations, the ability of tPA-DPN to mitigate the neurological side effects commonly associated with conventional tPA administration was evaluated in vivo using a severe middle cerebral artery occlusion/reperfusion (MCAO) mouse model. Behavioral and survival analyses demonstrated the beneficial effects of tPA conjugation to DPN compared with administration of free tPA alone (Fig. 5A). Histological assessment of lesion size and immunoglobulin G (IgG) extravasation, further corroborated by magnetic resonance imaging (MRI), revealed that the improved behavioral outcomes and survival rates were associated with reduced infarct size and decreased BBB leakage (Fig. 5C).

Mice treated with free tPA displayed severely impaired survival, activity, and neurological scores 24 h after occlusion. Most animals did not survive beyond the first day post-stroke, and the few surviving mice exhibited severe neurological deficits. In contrast, mice treated with tPA-DPN showed survival rates and behavioral scores comparable to those observed in saline-treated controls. These findings were further supported by histological analyses and MRI data demonstrating smaller ischemic lesions and lower BBB permeability in mice receiving tPA-DPN compared with free tPA. Importantly, these tissue-level observations were also consistent with cellular analyses performed on microglia and astrocytes.

In a previous study, Colasuonno et al. demonstrated that enhanced thrombolytic efficacy could be achieved with tPA-DPN containing only 1mg/kg of tPA in mice (Colasuonno et al. 2018). By comparison, the standard clinical dose of Alteplase^®^ is 0.9 mg/kg in humans, which corresponds to approximately 10 mg/kg in mice after body surface area normalization, a dose routinely employed in preclinical stroke studies (Nair and Jacob 2016). These findings suggest that tPA-DPN can achieve comparable, or even superior, therapeutic efficacy using approximately one-tenth of the conventional murine equivalent dose, highlighting the marked improvement in thrombolytic efficiency afforded by DPN-mediated drug delivery.

In addition to their enhanced thrombolytic activity, tPA-DPN significantly reduced the well-established adverse effects associated with intravenous tPA administration during the acute phase of ischemic stroke (Fig. 5E). This protective effect was maintained even when tPA-DPN were administered at the conventional murine dose of 10 mg/kg^12^, demonstrating that conjugation of tPA to DPN markedly attenuates the cerebral neurotoxic complications associated with free tPA treatment while preserving its therapeutic efficacy.

Moreover, the presence of tPA on the particle surface enhances particle adhesion to the clot due to the strong affinity between tPA and fibrin, as well as the possibility of establishing multivalent interactions between fibrin molecules within the clot and multiple tPA molecules exposed on the DPN surface.

**Fig. 6:**
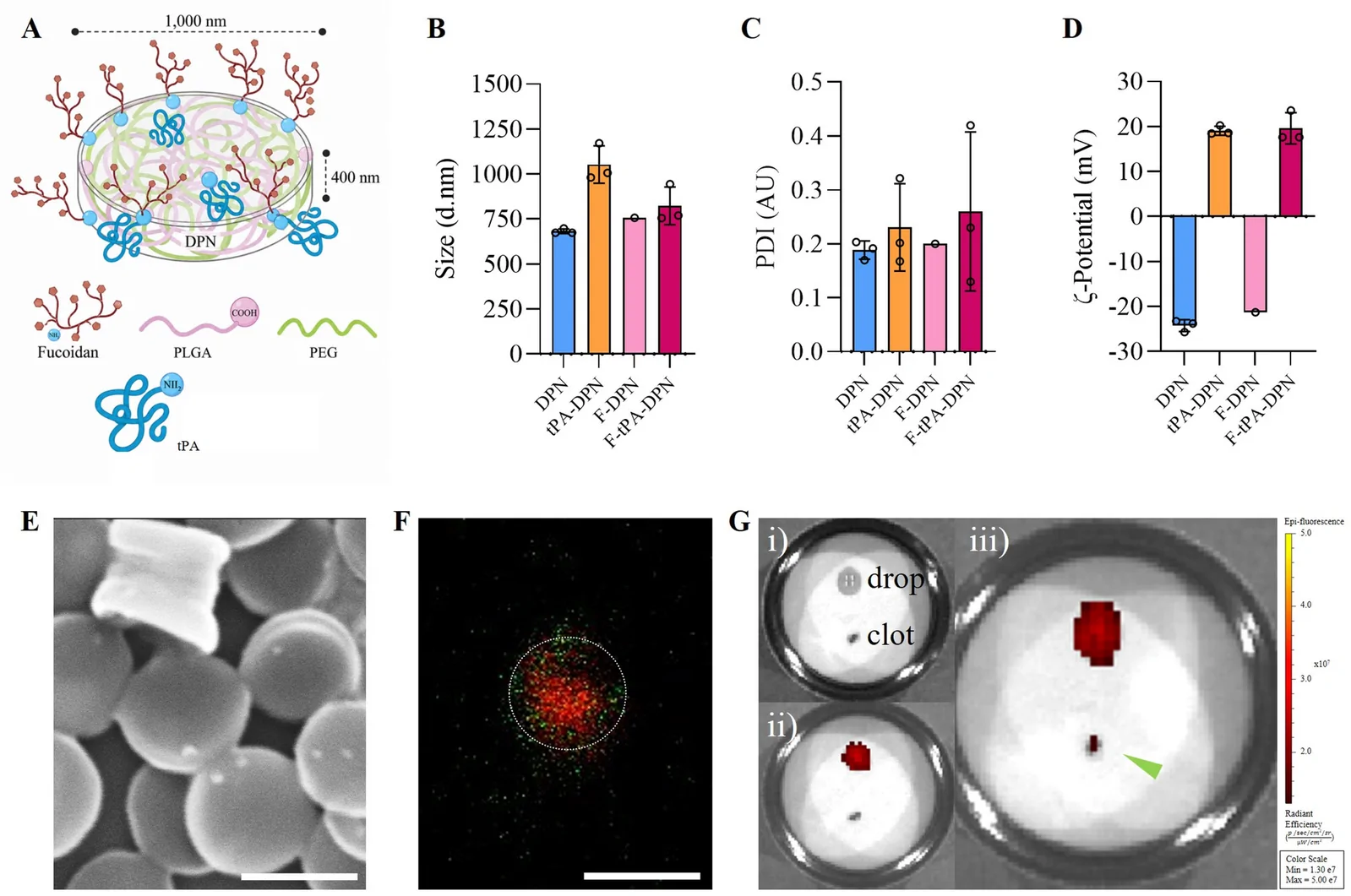
Improving clot selectivity and targeting efficiency through fucoidan particle decoration. A. Schematic representation of fucoidan-decorated tPA-DPN (F-tPA-DPN). **B–D**. Size, polydispersity index (PDI), and surface charge (ζ-potential) of the different particle formulations. **E**. Representative scanning electron microscopy image showing the morphology of F-tPA-DPN (scale bar: 1 μm). **F.** Confocal microscopy image of a single F-tPA-DPN highlighting the surface distribution of fucoidan (green) on rhodamine-labeled tPA-DPN (scale bar: 1 μm). **G**. Fluorescence imaging by IVIS following an in vitro binding assay. A small PBS droplet containing fluorescently labeled particles was placed in contact with an artificial clot and subsequently removed. No residual signal was detected on the clot after exposure to PBS alone (i) or tPA-DPN (ii), whereas a persistent fluorescent signal remained on clots exposed to F-tPA-DPN (iii, arrow), indicating enhanced clot-binding capability

To further improve clot selectivity and targeting efficiency, additional targeting ligands have also been investigated, including fucoidan. Fucoidan is a sulfated polysaccharide characterized by a strong and selective affinity for P-selectin (Tylawsky et al. 2023), a molecule highly expressed on activated platelets and endothelial cells during thrombotic events (Zhang et al. 1998). Fucoidan has been extensively explored in different nanomaterial-based delivery systems to improve the specificity and efficacy of thrombolytic therapies (Taille et al. 2025, Fournier et al. 2023, Zenych et al. 2021). A modified fucoidan, kindly supplied from the group of Chauvierre Cedric at Laboratory for Vascular Translational Science (LVTS), in INSERM and Université Paris Cité, Université Sorbonne Paris Nord, was combined with tPA-DPN technology. The modified fucoidan was carrying a terminal NH₂ group enabled its conjugation onto DPN through the same EDC/NHS bioconjugation strategy previously adopted for tPA functionalization. This configuration offers the potential advantage of combining the thrombolytic activity of tPA with the enhanced specificity provided by fucoidan-mediated targeting (Fig. 6A).

Preliminary investigations demonstrated that varying the tPA/fucoidan ratio during the bioconjugation reaction generated particles with distinct surface charges, ranging from positive (100% tPA) to negative (100% fucoidan). Fucoidan-functionalized tPA-DPN (F-tPA-DPN) maintained morphology, size, and shape comparable to those of conventional tPA-DPN and bare DPN, as confirmed by dynamic light scattering (DLS) and scanning electron microscopy (SEM) analyses (Fig. 6B). Successful fucoidan conjugation was further verified by confocal microscopy, which enabled visualization of the labeled polysaccharide on the discoidal particle surface (Fig. 6F).

Finally, F-tPA-DPN were evaluated in vitro by fluorescence imaging (Fig. 6G). In these experiments, F-tPA-DPN exhibited stable binding to artificial clots, whereas conventional tPA-DPN showed insufficient affinity to remain attached after transfer and washing procedures. These findings suggest that fucoidan functionalization significantly enhances clot-targeting capability.

Overall, the observed therapeutic performance can be attributed to the combined physicochemical and biological properties of DPN, including their size, discoidal shape, deformability, and adhesive interactions with fibrin. Their discoidal morphology and micrometric dimensions favor lateral drift within blood flow, increasing the probability of contact with vascular walls and thrombotic regions, thereby favoring interactions with both the endothelium and thrombi. In addition, the Young’s modulus of DPN, ranging from a few tens to a few hundreds of kPa and comparable to that of biological cells, may facilitate particle trapping and accumulation within the fibrin network.

Future studies should evaluate this strategy in additional stroke models and in the presence of clots with different biochemical compositions and mechanical properties. Furthermore, considering the continuous clinical development and approval of novel thrombolytic agents, incorporation of next-generation drugs such as Tenecteplase may further improve both therapeutic efficacy and safety profiles.

## Current limitations and challenges for clinical translation

Despite their promising preclinical performance, several challenges must be addressed before DPN can progress toward clinical translation. First, the template-assisted, top-down fabrication process, although offering precise control over particle geometry and mechanical properties, must be adapted to large-scale manufacturing while preserving production efficiency and consistent particle recovery. Unlike bottom-up self-assembly methods, which can often be adapted to large-volume or continuous-flow production, template-assisted fabrication involves several sequential operations, including template preparation, cavity filling, removal of excess material, particle solidification, release, recovery, and purification. Scaling up these steps requires not only an increase in template area or the parallelization of multiple templates but also preservation of uniform cavity filling and consistent particle recovery across the entire production system. Variations in polymer-paste rheology, solvent evaporation, applied pressure, drying conditions, could affect particle dimensions, stiffness, drug loading, and release kinetics, thereby introducing batch-to-batch variability. Moreover, repeated template fabrication and use may increase manufacturing time, cost, and the risk of defects or contamination. Translation of this approach will therefore require scalable and reproducible template-replication methods, greater process automation, standardized particle-harvesting procedures, and appropriate in-process monitoring under Good Manufacturing Practice (GMP) conditions. Continuous or roll-to-roll template-assisted manufacturing and highly parallelized production systems may provide potential routes toward higher throughput, although their feasibility for DPN and µCGP production remains to be demonstrated. On the other end, the authors have explored two-photon continuous flow lithography to produce microparticles. This technique might represent an alternative route to scale up non-spherical particles (Manghnani et al. 2022).

Robust quality-control procedures will also be required to ensure batch-to-batch reproducibility in terms of particle size, aspect ratio, stiffness, surface properties, drug loading, and release kinetics. In addition, suitable sterilization methods must be identified, as conventional approaches may alter polymer integrity, particle mechanics, or therapeutic payload stability. Long-term storage conditions and shelf life also remain to be established. Finally, although PLGA and PEG have a well-established history of biomedical use, DPN must be evaluated as an integrated drug–device product. Their regulatory development will therefore require comprehensive characterization of the manufacturing process, degradation products, biodistribution, pharmacokinetics, immunogenicity, toxicity, and long-term safety. Addressing these issues through standardized manufacturing and quality-by-design strategies will be essential to determine the clinical feasibility of the platform.

## Conclusion and future perspective

The body of experimental evidence presented here establishes DPN as a genuinely differentiated nanomedicine platform.

By systematically engineering key physical determinants of particle behavior, including size, shape, and deformability, rather than relying exclusively on surface chemistry or targeting ligands, this technology provides a mechanistically grounded framework for drug delivery across multiple pathological conditions, including cancer theranostics and ischemic stroke. Table 2 summarizes the key characteristics of the different DPN configurations discussed in this review. Beyond their distinctive physico-mechanical properties, DPN have demonstrated a favorable safety profile in preclinical studies. Their PLGA/PEG-based composition, together with their deformability, contributes to reduced recognition and sequestration by the reticuloendothelial system and prolonged circulation time (Palange et al. 2017). In vitro studies demonstrated no detectable metabolic impairment in human umbilical vein endothelial cells (HUVECs), (Key et al. 2015; Colasuonno et al. 2018), while in vivo investigations showed no significant alterations in inflammatory cytokines (IL-6, IL-10, and TNF-α) or biochemical markers of liver and kidney function, including AST, ALT, and creatinine (Key et al. 2015). Furthermore, repeated intravenous administration of DPN in murine cancer models was not associated with significant acute toxicity or behavioral alterations. Taken together, these findings indicate that DPN combines favorable biodistribution and prolonged vascular persistence with encouraging preclinical biocompatibility.

**Table 2:** Overview of the key characteristics of all DPN configurations

Technology	Main feature	Loading modality	Molecule loaded
Multi-passage DPN	Increased drug loading capacity	Direct	DTXL
Prodrug-loaded DPN	Modulate drug release	Absorption	DTXL
μCGP	Hierarchical system, payload versatility	Direct	Multiple
tPA-DPN	Improved vascular drug retention	Bioconjugation	tPA
Fucoidan-functionalized DPN	Improved targeting capability	Bioconjugation	tPA, Fucoidan

Nevertheless, longer-term investigations are required before definitive conclusions can be drawn regarding the safety and translational potential of DPN- and µCGP-based treatments. Future studies should comprehensively characterize their pharmacokinetics, biodistribution, metabolism, and clearance following both single and repeated administrations. Particular attention should also be given to chemically modified therapeutic agents, such as PEG–docetaxel conjugates, since conjugation may substantially alter drug solubility, protein binding, tissue distribution, cellular uptake, metabolism, and elimination compared with the unconjugated drug.

Looking forward, computational and machine-learning approaches could further support the rational development of these platforms by identifying relationships between physicochemical parameters and biological performance, guiding the optimization of particle size, shape, deformability, and formulation prior to experimental synthesis and validation. Such approaches could help reduce the experimental design space and accelerate the identification of formulations with favorable pharmacokinetic and therapeutic properties (Basak 2026, Basak 2026, Francesco et al. 2023).

Overall, the ability to independently modulate the physico-mechanical and surface properties of DPN supports their potential as a versatile platform for systemic drug delivery. The application of vascular-confined DPN to thrombolytic therapy provides a particularly compelling example: conjugation of tPA to the particle surface may preserve thrombolytic efficacy while limiting extravascular drug exposure, thereby reducing the neurotoxic and hemorrhagic complications associated with conventional systemic tPA administration. Together, these findings provide a strong rationale for further preclinical development of DPN-based therapies while highlighting the pharmacological, toxicological, manufacturing, and regulatory challenges that must be addressed before clinical translation.

## Data Availability

No datasets were generated or analysed during the current study.
